# Draft Genome Sequence of a Drug-Susceptible New Zealand Isolate of *Mycobacterium tuberculosis* Lineage 3

**DOI:** 10.1128/genomeA.00499-15

**Published:** 2015-05-21

**Authors:** Micheál Mac Aogáin, James E. Bower, Indira Basu, Joshua T. Freeman, Ronan F. O’Toole

**Affiliations:** a Department of Clinical Microbiology, School of Medicine, Trinity College Dublin, St. James’s Hospital, Dublin, Ireland; b LabPlus, Auckland City Hospital, Auckland, New Zealand; c Breathe Well NHMRC Centre of Research Excellence, School of Medicine, University of Tasmania, Hobart, Tasmania, Australia

## Abstract

We report here the draft whole-genome sequence of a drug-susceptible lineage 3 (East-African Indian) isolate of *Mycobacterium tuberculosis* from New Zealand (NZ3DS1) and compare it to a multidrug-resistant lineage 3 isolate (NZ3MDR1) with an identical 24-locus mycobacterial interspersed repetitive-unit–variable-number tandem-repeat profile.

## GENOME ANNOUNCEMENT

Tuberculosis (TB) is a leading cause of infectious mortality worldwide, killing approximately 1.5 million people each year (1). In earlier work, we determined that 17.7% (95% CI, 14.5% to 21.3%) of TB cases in New Zealand during 2010 and 2011 were caused by lineage 3 (East-African Indian, including CAS) of *Mycobacterium tuberculosis* complex (2), a predominant lineage on the Indian subcontinent (3, 4). Subsequently, we described a multidrug-resistant (MDR) isolate (NZ3MDR1) from New Zealand that belonged to lineage 3 (5). Of interest was the isolation of a New Zealand lineage 3 with a 24-locus mycobacterial interspersed repetitive-unit–variable-number tandem-repeat (MIRU-VNTR) profile identical to that of to NZ3MDR1 but which was fully susceptible to streptomycin, isoniazid, rifampin, ethambutol, and pyrazinamide.

Here, genomic DNA of the latter drug-susceptible lineage 3 isolate, NZ3DS1, was sequenced using an Illumina MiSeq instrument. A total of 3,413,992 paired-end reads were mapped to the *M. tuberculosis* strain H37Rv reference genome (accession no. AL123456.3) by Burrows-Wheeler alignment (6). This yielded an average read depth of 78-fold, covering 98.6% of the H37Rv genome. A consensus sequence was called using the SAMtools analysis suite (7), yielding a 4,294,796-bp draft assembly of 158 contigs.

Single-nucleotide variant (SNV) annotation was performed using snpEff (8), which identified a total of 1,330 SNVs in the assembled NZ3DS1 genome with respect to H37Rv, of which 723 were nonsynonymous. Of the 1,330 SNVs identified in NZ3DS1, 1,262 were also present in NZ3MDR1, while the two strains differed by 126 SNVs. A nonsynonymous mutation was identified in the gene Rv1908c (*katG*) [cGg/cTg, R463L]; however, this mutation was reportedly detected in isoniazid-sensitive strains of *M. tuberculosis*, indicating low specificity for isoniazid resistance (9). In terms of other first-line drugs, nonsynonymous mutations were detected in the gene Rv3793 (*embC*) [cGg/cAg, R738Q; atG/atA, M800I], although their specificities with respect to ethambutol resistance remain to be established. Interestingly, the latter M800I mutation was absent from NZ3MDR1. Nonsynonymous mutations were not detected with respect to genes associated with pyrazinamide resistance, i.e., Rv2043c (*pncA*), Rv1630 (*rpsA*), and Rv3601c (*panD*) (10). Regarding second-line drug resistance, SNVs were identified in Rv0006 (*gyrA*) [Gag/Cag, E21Q; aGc/aCc, S95T; gGc/gAc, G668D]. The S95T mutation, in particular, has been demonstrated to have low specificity with respect to fluoroquinolone resistance (11).

Although the NZ3DS1 and NZ3MDR1 cases occurred in Indian-born individuals, as with most cases involving lineage 3 in New Zealand (2), there are no known epidemiological links between the two cases which occurred a year apart. One cannot exclude the possibility of contact within New Zealand or prior arrival in New Zealand; however, the number of SNV differences between NZ3DS1 and NZ3MDR1 would not appear to support these possibilities. No further cases with the same MIRU-24 pattern have been recorded in New Zealand. That the two isolates, NZ3MDR1 and NZ3DS1, had the same MIRU-24 profile but different drug-susceptibility profiles indicates an irregular correlation between MIRU-24 genotype and phenotypic drug susceptibility. Recently, it was reported that MIRU loci could predict the drug resistance of *M. tuberculosis* (12). In light of the above findings, some caution is needed with regard to the extrapolation of MIRU data for drug susceptibility determination.

### Nucleotide sequence accession number.

This whole-genome shotgun project has been deposited at DDBJ/EMBL/GenBank under the accession number JZDR00000000.

## References

[1] World Health Organization 2014 Global tuberculosis report. WHO, Geneva, Switzerland.

[2] Yen S, Bower JE, Freeman JT, Basu I, O’Toole RF 2013 Phylogenetic lineages of tuberculosis isolates in New Zealand and their association with patient demographics. Int J Tuberc Lung Dis 17:892–897. doi:10.5588/ijtld.12.0795. 23635796

[3] Gagneux S, DeRiemer K, Van T, Kato-Maeda M, de Jong BC, Narayanan S, Nicol M, Niemann S, Kremer K, Gutierrez MC, Hilty M, Hopewell PC, Small PM 2006 Variable host-pathogen compatibility in *Mycobacterium tuberculosis*. Proc Natl Acad Sci USA 103:2869–2873. doi:10.1073/pnas.0511240103. 16477032 PMC1413851

[4] Reed MB, Pichler VK, McIntosh F, Mattia A, Fallow A, Masala S, Domenech P, Zwerling A, Thibert L, Menzies D, Schwartzman K, Behr MA 2009 Major *Mycobacterium tuberculosis* lineages associate with patient country of origin. J Clin Microbiol 47:1119–1128. doi:10.1128/JCM.02142-08. 19213699 PMC2668307

[5] Mac Aogáin M, Johari BM, Bower JE, O’Toole RF 2014 Draft genome sequence of a multidrug-resistant New Zealand isolate of *Mycobacterium tuberculosis* lineage 3. Genome Announc 2(5):e01017-14. doi:10.1128/genomeA.01017-14. 25323711 PMC4200149

[6] Li H, Durbin R 2010 Fast and accurate long-read alignment with Burrows-Wheeler transform. Bioinformatics 26:589–595. doi:10.1093/bioinformatics/btp698. 20080505 PMC2828108

[7] Li H, Handsaker B, Wysoker A, Fennell T, Ruan J, Homer N, Marth G, Abecasis G, Durbin R 2009 The Sequence Alignment/Map format and SAMtools. Bioinformatics 25:2078–2079. doi:10.1093/bioinformatics/btp352. 19505943 PMC2723002

[8] Cingolani P, Platts A, Wang le L, Coon M, Nguyen T, Wang L, Land SJ, Lu X, Ruden DM 2012 A program for annotating and predicting the effects of single nucleotide polymorphisms, SnpEff: SNPs in the genome of *Drosophila melanogaster* strain w1118; iso-2; iso-3. Fly (Austin) 6:80–92. doi:10.4161/fly.19695. 22728672 PMC3679285

[9] Ping W 2011 Molecular detection of *M. tuberculosis* complex in clinical lung tissue samples. Ph.D. thesis Beijing Union Medical College, Beijing, China.

[10] Köser CU, Bryant JM, Becq J, Török ME, Ellington MJ, Marti-Renom MA, Carmichael AJ, Parkhill J, Smith GP, Peacock SJ 2013 Whole-genome sequencing for rapid susceptibility testing of *M. tuberculosis*. N Engl J Med 369:290–292. doi:10.1056/NEJMc1215305. 23863072 PMC3836233

[11] Rodwell TC, Valafar F, Douglas J, Qian L, Garfein RS, Chawla A, Torres J, Zadorozhny V, Kim MS, Hoshide M, Catanzaro D, Jackson L, Lin G, Desmond E, Rodrigues C, Eisenach K, Victor TC, Ismail N, Crudu V, Gler MT, Catanzaro A 2014 Predicting extensively drug-resistant *Mycobacterium tuberculosis* phenotypes with genetic mutations. J Clin Microbiol 52:781–789. doi:10.1128/JCM.02701-13. 24353002 PMC3957771

[12] Yu-Feng W, Chao J, Xian-Feng C 2015 Drug-resistant tuberculosis can be predicted by mycobacterial interspersed repetitive unit locus. Front Microbiol 6:147. doi:10.3389/fmicb.2015.00147. 25759689 PMC4338821

